# Micro-Expressions of Fear During the 2016 Presidential Campaign Trail: Their Influence on Trait Perceptions of Donald Trump

**DOI:** 10.3389/fpsyg.2021.608483

**Published:** 2021-06-02

**Authors:** Patrick A. Stewart, Elena Svetieva

**Affiliations:** ^1^Department of Political Science, University of Arkansas, Fayetteville, AR, United States; ^2^Department of Communication, University of Colorado Colorado Springs, Colorado Springs, CO, United States

**Keywords:** micro-expression, Facial Action Coding System (FACS), political speeches, 2016 presidential election, competence, trustworthiness, Donald Trump, Hillary Clinton

## Abstract

The 2016 United States presidential election was exceptional for many reasons; most notably the extreme division between supporters of Donald Trump and Hillary Clinton. In an election that turned more upon the character traits of the candidates than their policy positions, there is reason to believe that the non-verbal performances of the candidates influenced attitudes toward the candidates. Two studies, before Election Day, experimentally tested the influence of Trump’s micro-expressions of fear during his Republican National Convention nomination acceptance speech on how viewers evaluated his key leadership traits of competence and trustworthiness. Results from Study 1, conducted 3 weeks prior to the election, indicated generally positive effects of Trump’s fear micro-expressions on his trait evaluations, particularly when viewers were first exposed to his opponent, Clinton. In contrast, Study 2, conducted 4 days before Election Day, suggests participants had at that point largely established their trait perceptions and were unaffected by the micro-expressions.

## Introduction

The 2016 United States presidential election will likely stand out as one of the most unusual, if not aberrant, of campaigns in American history (e.g., Cavari et al., 2017; Senior(ed.), 2018). It featured a long-standing Washington D.C. insider and first female presidential nominee for a major political party–Democratic Party candidate Hillary Clinton–being challenged by reality television star and political neophyte–the Republican Party candidate Donald Trump–for the presidency. With this historical first, what stood out was the controversial nature of each candidate, the polarized attitudes of their respective supporters and opponents, and ultimately the divisive effect of their presidential campaigns.

In an election campaign that featured less policy debate than an examination of the trustworthiness of the two primary candidates, there is reason to believe that the non-verbal performances of candidates influenced followers’ perceptions of their respective leadership capacity. Indeed, in competitive settings such as elections, followers and prospective followers may derive more reliable evaluative information concerning candidates’ potential future performance as a leader from their non-verbal behavior in conjunction with verbal utterances than from policy statements alone (Sullivan and Masters, 1988; Stewart et al., 2009a; Van Vugt and Ahuja, 2011). More specifically, the coherence of non-verbal delivery with verbal statements likely affects the credibility and appropriateness of candidate statements (Beattie, 2016) and, in turn, affect perceptions of the leadership traits held by the candidates.

Several decades of research in social perception and neuroscience has suggested that even the subtle and/or fleeting non-verbal behavior of an individual can influence how they are perceived (Ambady and Rosenthal, 1992; Olivola and Todorov, 2010). Moreover, this influence is often outside the realm of conscious awareness or deliberation (Hassin et al., 2005). This behavior might be so slight and elusive as to not be cognitively attended to, or lead to emotional response that is more of a “gut feeling” (Niedenthal, 1990; Svetieva and Frank, 2016) with viewers engaging in non-heuristic, effortful information processing only after interpreting behavior as inappropriate (Bucy and Newhagen, 1999). While not common, research shows that the facial displays of leaders lasting less than half a second, i.e., a micro-expression, may communicate anxiety, sadness, and even delight (Porter and ten Brinke, 2008; Stewart et al., 2009b; ten Brinke and Adams, 2015).

With this in mind, the research carried out here identifies and tests the influence of subtle and fleeting facial expressions by Donald J. Trump during his 2016 Republican National Convention nomination acceptance speech. We utilize a minute-long clip during which Trump emphasizes the threat from terrorism by listing recent attacks in San Bernardino and at the Boston Marathon, a point during which he also displays very brief micro-expressions of fear. The relatively rare occurrence of these micro-expressions during a public speech by a political figure allow us an opportunity to experimentally test their influence on viewers, including perceptions of Trump’s leadership and the basic trait dimensions of competence and warmth.

This paper is organized to first define micro-expressions and their influence on perceivers before considering more generally the influence of non-verbal behavior on response to political figures and perceptions of leadership traits by followers. We characterize the micro-expressions by Donald Trump though the use of the Facial Action Coding System (FACS) and identify the emotions displayed using the EMFACS (Emotion FACS) emotion dictionary and the Componential Processing Model (CPM) of emotion appraisal. From there, we analyze the findings of two experimental studies carried out in the final days of the 2016 presidential election. The first study, implemented 3 weeks prior to Election Day, utilized an opportunity sample of university and community college students to test the influence of micro-expressions (by removing their occurrence). Three different conditions were used: (1) original micro-expression-intact (ME-intact) condition, featuring the original, unaltered clip, (2) the micro-expression-removed (ME-removed) condition, where the frames containing the micro-expressions were edited out of the video, and (3) a micro-expression-control (ME-control) condition, where an equivalent number of frames were removed from another point in the clip, to test any possible confounds of having an edit-point in the footage. To provide for comparison and understand the contrast effects derived from his particular opponent, we chose a comparable video from Hillary Clinton’s Democratic National Convention nomination speech with her addressing the threat of terrorism. The second study replicated the first study with Trump and Clinton supporters drawn from a nationally representative sample 4 days prior to the election.

### Political Leadership and Non-verbal Behavior

Starting with the groundbreaking work carried out by an interdisciplinary team of scholars in the 1980s, social scientists have appreciated the role played by the non-verbal behavior of politicians on follower preference and support. The “Dartmouth Group” (McHugo et al., 1985; Masters et al., 1986; Sullivan and Masters, 1988) and its adherents (Bucy, 2000; Salter, 2007; Dumitrescu et al., 2015) considered interpersonal interactions based upon both social rank (dominant or submissive) and type of behavior (competitive or affiliative), and in doing so they examined facial display behavior according to basic emotions theory (e.g., anger-threat, fear-submission, happiness-reassurance, and sadness-appeasement).

Theoretical and methodological advances in the objective measurement of facial behavior enabled researchers to identify how even subtle facial displays can influence perceptions of the emotion felt and behavioral intent of the sender (Trichas and Schyns, 2012; Stewart and Ford Dowe, 2013; Stewart et al., 2015a). Indeed, research considering the response to a presidential “rally-round-the-flag” speech in which leaders address followers regarding an external threat suggests display behaviors that last close to or less than one-half a second can influence emotions felt by viewers (Stewart et al., 2009b; Brand, 2012).

### Micro-Expressions

Micro-expressions, as facial displays of emotion that occur on the face for a fraction of a second (Ekman and Friesen, 1969), are one instance of subtle expressive behavior. Unlike typical facial expressions of emotion (which last up to 4 s), they are hypothesized to be produced largely involuntarily, and may indicate otherwise concealed emotions and behavioral intent (Porter and ten Brinke, 2008; Frank and Svetieva, 2015). Although micro-expressions are thought to be too fleeting to be consciously noticed by laypersons, there is evidence that they do have an implicit communicative impact (Svetieva and Frank, 2016). For example, Stewart et al. (2009b) found that when George H. W. Bush displayed micro-expressions indicating anger, disgust, and happiness during his August 8, 1990 nationally televised speech prior to the United States engaging in the first Gulf War, they had the effect of decreasing the speech’s persuasive impact. While this study illuminated the potential impact of micro-expression displays during a “real world” example with grave political and societal implications, limitations remain; the varied emotional and behavioral intent leaked by the seven micro-expressions during the nearly 12 min speech, the significant time lag between when the study was carried out and when the events occurred, and the focus solely on felt emotions. We address some of these limitations in the present study and address the key question of whether viewers will be influenced by micro-expressions by putative candidates in a contemporaneous electoral situation. In other words, by considering contextual information during an ongoing presidential campaign, we can obtain greater understanding regarding individual response to brief, subtle non-verbal signals, as well as the various factors influencing their interpretation (Fridlund, 1994, 2017; Mehu and Scherer, 2012; Hess and Hareli, 2017; Scherer et al., 2017).

However rehearsed the candidates are, public, televised speeches offer insights into the candidates by virtue of how they address their supporters. Non-verbal behavior during these speeches has the potentially to affect electoral outcomes by increasing enthusiasm amongst the base, or alternately diminishing support through a poor performance. Even when candidates rely upon prepared and vetted scripts, valuable emotional and behavioral information is revealed through their non-verbal behavior, which in turn impacts inferences of their leadership traits (Bucy, 2000; Lancaster et al., 2013; Gong and Bucy, 2015).

### Communicating Competence and Trustworthiness Through Non-verbal Cues and Signals

There is clear evidence that social behavior is evaluated into two major dimensions, whether based upon trait evaluations, broadly defined as competence and warmth/trustworthiness (Fiske et al., 2007) or, when considering non-verbal behavior, as serving the social ends of dominance and affiliation, respectively (Masters et al., 1986; Hareli et al., 2015; Stewart et al., 2015b). Likewise, the two trait dimensions are ones that political figures have been evaluated on (Kinder et al., 1980; Abelson et al., 1982), even though these dimensions may be valued differently based upon the perceived strengths of a political party’s candidate (Cornwell et al., 2015) or the contextual requirements of the audience (D’Errico, 2019).

Additionally, the personality traits of political figures may be inferred swiftly based upon minimal information, including facial physiognomy (Todorov et al., 2005) and body movement (Koppensteiner et al., 2016), with evidence that this minimal information also drives leadership preferences (Vigil, 2010; Laustsen and Petersen, 2016). Perhaps more pertinent for the candidates themselves, trait perceptions may change in response to unmediated electoral events such as the presidential debates held in the weeks before Election Day (Patterson et al., 1992; Wicks, 2007). Even short video clips of non-verbal behavior of political leaders during televised news stories influence trait attributions (Sullivan and Masters, 1994; Bucy, 2000).

Judgments of trustworthiness, related to the warmth trait dimension, are particularly salient for political figures. Congruence between emotions displayed and verbal content are crucial for such trait attributions, such that the “discrepancy between the emotional cues displayed and the assumed emotional experience associated with the event is taken as a possible indicator that the content of symbolic signals should not be trusted.” (Mehu and Scherer, 2012 p. 402). The importance of multi-modal non-verbal congruity in the messaging by political leaders is increasingly appreciated by scholars; recent years have seen computer vision used to describe political figures from multiple nations and their ability to communicate intent to their followers effectively (D’Errico and Poggi, 2019; Kang et al., 2020).

Micro-expressions, as instances of unintended emotional “leakage,” may by definition be considered incongruous displays. However, their appropriateness needs to be evaluated in the context of the larger communicative act. Indeed, they may be perceived, consciously or preconsciously, as inappropriate emotional leakage (Stewart et al., 2009b) but they also may exist as more appropriate non-verbal “punctuation” of verbal statements (Stewart, 2010), one that bolsters the perceived competence and/or trustworthiness of a speaker. In the present study, we examine how micro-expressions of fear during a speech enumerating the blight of terrorism influence both trustworthiness and competence trait evaluations broadly. We also hypothesize that competence will be affected to a greater extent than trustworthiness due to the close relationship between fear felt from a threat and the perceived competence of the leader to address it.

### The Influence of Followership on Leader Evaluation

Responses to candidate facial display behavior may be accentuated or attenuated depending on the relationship between the contender and their observer. Followers are more likely to have an appropriate and stronger response to their leader than will those in opposition or non-aligned. For instance, when evaluating different types of smiles displayed by President Obama, supporters perceived greater happiness encoded in his smiles than did either his opponents or disinterested third parties (Stewart and Ford Dowe, 2013). Political identity plays an important role, with party identification leading to higher positive and lower negative emotional response to the preferred party’s candidate display behavior as compared with the other party’s candidate (Sullivan and Masters, 1988; McHugo et al., 1991; Way and Masters, 1996; Bucy and Newhagen, 1999). However a more direct measure of support is voting. Whether the intention to vote, or having voted for a candidate, this activity is a fundamental indicator of support and followership. Specifically, it has been long understood that the candidates of the major political parties, not necessarily the parties themselves or the political ideology they presumably represent, are a major spur to political activity.

The current political climate, with its strong bipartisan divisions, renders the influence of followership even more salient. Both candidates have been characterized as controversial and divisive, so much so that a vote for one is a strong protest vote against their opponent. For this reason, in addition to examining followership, we utilize a clip of Hillary Clinton to “contextualize” how viewers perceive Donald Trump and, specifically whether the impact of his micro-expressions is moderated by the context in which they are viewed (i.e., after viewing a similarly themed speech by Hillary Clinton).

### Focal Footage

A central refrain throughout Donald Trump’s 2016 presidential campaign was the threat posed by terrorism, whether domestic or international. This theme was the central emotional touchstone of his Republican National Convention acceptance speech, a 75 min long address, that also happened to be one of the very few speeches for which Trump stayed on script and used teleprompters. Midway through, and while elaborating on the threat posed by ISIS by listing attacks (see Box 1), Trump displayed two micro-expressions associated with the perception of threat and, concomitantly, experiencing fear. The minute-long segment was independently coded by the authors (both FACS certified coders) who agreed on the exact timing (within three video frames) and form (in action units displayed) of each micro-expression.

**Stimuli from Donald Trump’s RNC Nomination Acceptance Speech.**

Donald Trump (44/45s; 1323/1350f) “(*0–4s applause*) Once again, France is the victim of brutal Islamic terrorism (*ME-control edit 1–17f [261-288]*). Men, women, and children viciously mowed down. Lives ruined. Families ripped apart. A nation in mourning (*ME-control edit 2–10f [625-635]*). The damage and devastation that can be inflicted by Islamic radicals has been over and over–at the World Trade Center, at an office party in San Bernardino (*ME-removed edit 1–17f [1028-1045]*), at the Boston Marathon (*ME-removed edit 2–10f [1100-1110]*), and a military recruiting center in Chattanooga, Tennessee. And many, many other locations.”

Throughout the chosen clip, while Trump’s eye blink rate was comparatively low (21.3 blinks per minute) suggesting a relative lack of anxiety, he made extensive use of hand and arm movements to underscore his statements (Bull, 2003). With this clip he alternates between “beats” with his right hand for part of the clip, switching to his left hand before placing both hands on the podium while listing four locations in the United States that were attacked by terrorists. After the sequential listing of “an office party in San Bernardino” and “at the Boston Marathon,” Trump displayed moderately strong lip stretches (AU 20), along with his lips parting (AU 25) for both micro-expressions. The first micro-expression (see Supplementary Figure 1), while longer at just over a half a second (17 frames) and involving a sharp intake of breath, involved fewer and arguably subtler muscular movements when compared to the second micro-expression. This latter display not only involved a strong lip stretch (AU 20) and lips parting (AU 25), but also saw Trump’s jaw drop (AU 26) and thrust forward (AU 29), displaying the lower teeth all the way to their roots over the course of this less than half second (10 frames) clip.

The lip stretches (AU 20) that occur in both clips result from the action of the *Risorius* muscle, a facial display that pulls the lip corners back toward the ears and is reliably linked to the experience of fear, possibly as an action that prepares the sender to vocalize loudly (Ekman and Friesen, 2003; Mehu et al., 2011). This display is accentuated by the involvement of the lip depressor that results in the greater visibility of the white patch of lower teeth in the second micro-expression. These displays are “reliable” in the sense that it allows for accurate recognition of both the underlying emotional state and the social intent of the sender, Donald Trump (Ekman, 2003; Mehu et al., 2011).

## Study 1 Method

### Participants

Participants were recruited from three institutions of higher education (two universities and one community college) from across the state of Arkansas and were provided extra credit for their participation.

A total of 221 individuals participated in this study, which took place 3 weeks prior to the election (October 16–29, 2016). Of the 249 initial participants, the responses of 12 were removed for not completing the survey and a further 16 were removed for not responding to the attention/manipulation check question (“Please list some of the thoughts you had while watching the video clip:”).

Of the 221 participant responses retained for analysis, 61.5% identified as female, 78.3% identified as Caucasian (with 7.7% African-American, 3.2% Asian, 5.0% Hispanic, 1.4% Native American, and 4.5% other ethnicity), with a mean age of 23 (range 16–60, *SD* = 7.4). Participants identified themselves as either supporting Hillary Clinton (35.3%), Donald Trump (34.8%), or not voting/supporting other candidates (29.9%). We found no statistical differences in the demographic profiles (sex, ethnicity, age or vote) of participants assigned to the three experimental conditions.

### Materials and Procedure

Prior to the treatment, participants were asked basic demographic information (age, sex, ethnicity), questions about whether they were registered to vote, the political party they identify with and the extent of their identification, their political ideology, and finally, what candidate they are most likely to vote for in the upcoming 2016 election. Participants were then randomly assigned in a two order (Trump video first, Clinton video first) by three micro-expression (ME-intact, ME-removed, ME-control) condition.

Immediately after the video clips were viewed, participants were asked to evaluate candidate leadership traits based upon how sincere, aggressive, strong, active, competent (competence dimension), as well as how intelligent, caring, trustworthy, agreeable, and warm (warmth dimension) they appeared during the short video clips. Responses ranged from “Not at all” to “Extremely” on a seven-point (0–6) scale, with the additive scales having a thirty-point range. Trump’s competence (α = 0.82, *M* = 16.20, *SD* = 7.57) and warmth (α = 0.94, *M* = 11.58, *SD* = 8.81) scales exhibit good-to-excellent reliability.

## Study 1 Results

Findings suggest that the experimental treatment had a significant and weak-to-moderate main effect on ratings of Trump’s trait competence, *F* (2, 203) = 3.940, *p* = 0.021, ηρ^2^ = 0.037, as did the interaction of the order of presentation with the treatment, *F* (1, 203) = 3.528, *p* = 0.031, ηρ^2^ = 0.034. As expected, Trump’s micro-expressions of fear had an effect upon participant perception of his competence, with the ME-removed condition being significantly different from the ME-intact (*p* = 0.028) and ME-control condition (*p* = 0.009). Specifically, the absence of the fear displays in the ME-removed condition (*M* = 14.6; SE = 0.74) led to decreased perception of Trump’s competence by participants when compared to the conditions where they were present (ME-intact *M* = 16.91; SE = 0.74; ME-control *M* = 17.25; SE = 0.69).

**FIGURE 1:**
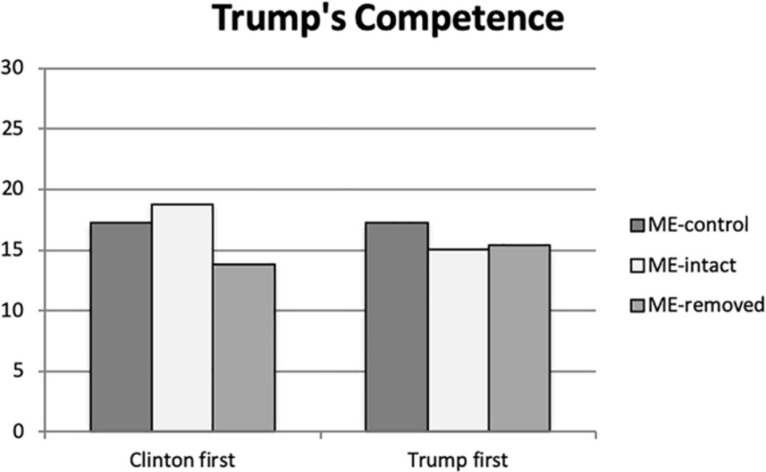
Donald Trump’s perceived competence by Study 1 participants.

Understanding the influence of Trump’s fear micro-expressions on his perceived competence is further elaborated upon when considering the interaction of the treatment with presentation order (see Figure 1). When Trump’s video was presented first, there was no significant difference based upon the treatment^1^; however, when Clinton’s clip was seen first by participants, significantly greater levels of competence was perceived as portrayed by Trump when he displayed micro-expressions of fear (ME-intact *p* = 0.001; ME-control *p* = 0.025).

As expected, who the respondent planned to vote for had a significant and large effect on evaluations of Trump, *F* (2, 203) = 60.461, *p* < 0.001, ηρ^2^ = 0.373. Trump supporters evaluated his competence as significantly higher (*M* = 22.22; SE = 0.71) than either Clinton supporters (*M* = 11.39; SE = 0.70) or those unaffiliated with either candidate (*M* = 15.13; SE = 0.75). On the other hand, the order of video presentation did not have a significant effect, *F* (1, 203) = 0.785, *p* = 0.377, ηρ^2^ = 0.004. When two-way interactions were considered, we found no significant effects for either order × vote, *F* (2, 203) = 0.980, *p* = 0.377, ηρ^2^ = 0.010, or for treatment × vote, *F* (4, 203) = 0.517, *p* = 0.723, ηρ^2^ = 0.010. Likewise, the three-way interaction of order × treatment × vote, *F* (4, 203) = 1.198, *p* = 0.31, ηρ^2^ = 0.023, was not significant.

**FIGURE 2:**
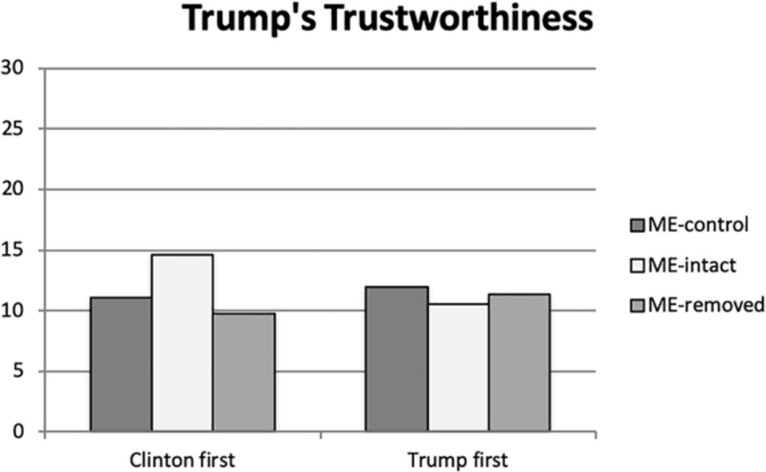
Donald Trump’s perceived trustworthiness by Study 1 participants.

Consideration of participant perceptions of Trump’s trustworthiness suggest that while neither the experimental micro-expression treatment, *F* (2, 203) = 1.912, *p* = 0.150, ηρ^2^ = 0.018, nor the order of presentation were significant, *F* (1, 203) = 0.452, *p* = 0.502, ηρ^2^ = 0.002, the interaction of these two variable had a significant and small-to-moderate effect, *F* (2, 203) = 4.293, *p* = 0.015, ηρ^2^ = 0.041. Here, participant’s perception of Trump’s trustworthiness shows a pattern similar to that seen with his perceived competence. Those viewing his video first were not affected by the presence or absence of the fear micro-expressions (see Figure 2). However, those exposed to the Clinton video first evaluate Trump’s trustworthiness as significantly greater when viewing the ME-intact video than the ME-removed video (*p* = 0.002) and the ME-control video (*p* = 0.017). While the former can be expected, especially as part of an overall evaluation of a clip, the significant difference between the ME-intact and the ME-control videos suggests that the slight discontinuities in the edited experimental videos may have negatively impacted perceived trustworthiness in the speaker.

Vote was highly significant and had a large effect on perceived trustworthiness, *F* (2, 203) = 113.045, *p* < 0.001, ηρ^2^ = 0.527. As was the case with competence, Trump’s supporters perceived more trustworthiness (*M* = 19.56; SE = 0.72) than did Clinton supporters (*M* = 4.47; SE = 0.71) or those not supporting either candidate (*M* = 10.60; SE = 0.76). However, when two-way interactions were considered, we found no significant effects for order × vote, *F* (2, 203) = 1.93, *p* = 0.376, η^2^ = 0.010, or for treatment × vote, *F* (4, 203) = 0.882, *p* = 0.476, η^2^ = 0.017, as was the case for the three way interaction of order × treatment × vote, *F* (4, 203) = 0.347, *p* = 0.846, η^2^ = 0.007.

## Study 1 Discussion

Our first and most salient finding is that Donald Trump’s micro-expressions of fear significantly affect perceptions of his competence both directly as a result of viewing them and when contextualized by viewing Hillary Clinton’s video first. In both cases, and as expected given the nature of the treatment, there was a small-to-moderate effect, with an increase in participant evaluation of this trait. Additionally, while trustworthiness was not expected to be directly affected by the micro-expressions, this trait was perceived as enhanced when the Clinton video was viewed first. In other words, not only did the two micro-expressions have the hypothesized effect, they apparently work as a form of non-verbal punctuation. The influence of this indicator of appraised threat was furthermore enhanced when candidate Trump was framed as being in a competitive context by having the Clinton clip viewed first, in turn affecting both of his leadership traits positively.

Hillary Clinton was also a candidate that was plagued with questions about her authenticity and trustworthiness. This was not only due to her prior political actions but her communicative style, which was often characterized as controlled (for example, Clinton’s speech did not contain any micro-expressions). Donald Trump on the other hand, pursued a communicative strategy that relied on more off-the-cuff remarks, and an unpracticed, unpolished delivery. In the present study, his emotional leakage proved beneficial in the context of his emotion-filled speech, especially when prefaced by footage of his more controlled, and less “authentic” opponent.

Methodologically, the inclusion of a control condition in which equivalent edits to non-micro-expression segments of the speech were carried out, enables us to conclude that micro-expressions specifically impact human communication rather than footage discontinuities.

Finally, the significant and rather large effect size of previously held candidate preferences on both of Trump’s trait perceptions is expected given not just the nature of followership, but also the charged nature of the 2016 election to that point. Trump followers saw their candidate as possessing both trait dimensions to a greater degree than did Clinton’s supporters, with the undecided and uncommitted midway between the two groups.

Given these findings, the opportunity to replicate this study during the historic 2016 election, and the open question as to whether closer proximity to Election Day accentuates or attenuates the influence of micro-expressions, we focused on the influence of the presence or absence of Trump’s micro-expressions on trait perceptions by his or Clinton’s supporters from across the United States. While it is possible that greater proximity to Election Day would lead to greater attention directed at the facial displays of both candidates (Sullivan and Masters, 1988), thus augmenting the influence of Trump’s micro-expressions, the same proximity could also mean that minds may have been made up concerning what traits both candidates possessed. Thus, we undertook Study 2 in the days immediately prior to the general election, using a sample of individuals that either supported Donald Trump or Hillary Clinton.

## Study 2 Method

### Participants

Study participants were recruited using a Qualtrics panel pool of opt-in participants from across the United States. A total of 212 individuals participated in this study, which took place 4 days prior to the election (November 4, 2016). Forty-one responses were removed for not responding with a substantive answer to the manipulation check question with an additional two removed from analysis due to not identifying either Trump or Clinton as their preferred candidate.

Of the remaining 169 participants, 68.8% identified as female, 81.8% identified as Caucasian (with 8% African-American, 2.8% Asian, 4.5% Hispanic, 1.1% Native American, and 1.7% other ethnicity), and the average age was 40 years old (range 18–84, SD = 15.1). The majority of participants identified themselves as supporting Donald Trump (50.6%) followed by those voting for Hillary Clinton (49.4%). When randomness in assignment was tested regarding the treatment or order conditions, we found no statistical bias (all *p*-values < 0.10) for sex, ethnicity, age, or vote.

### Measures

The same general approach was used to collect data from the United States sample. The only difference came with the experimental design in which were randomly assigned in a two order treatment (Trump or Clinton video first) as before by a two (ME-intact or ME-removed), instead of three, micro-expression treatment design.

Participant evaluation of Clinton and Trumps’ leadership traits followed the same pattern as with Study 1, with similar findings. Trump’s competence (α = 0.868, *M* = 16.97, SD = 8.46) and warmth (α = 0.97, *M* = 13.73, SD = 10.36) scales exhibit good-to-excellent reliability, as did Clinton’s competence (α = 0.89, *M* = 15.02, SD = 8.91) and warmth (α = 0.97, *M* = 12.89, SD = 10.17).

### Trump’s Competence and Trustworthiness

Unexpectedly, the micro-expression treatments’ effect on perceptions of Trump’s leadership trait of competence was not significant, *F* (1, 173) = 0.372, *p* = 0.543, ηρ^2^ = 0.002, although order of presentation did affect participant perceptions, *F* (1, 173) = 6.340, *p* = 0.013, ηρ^2^ = 0.037. Here, viewers seeing the Clinton video first evaluated Trump’s competence as being lower (*M* = 15.79; SE = 0.72) than did those who viewed the Trump videos first (*M* = 18.30; SE = 0.69).

As expected, who the respondent planned to vote for had a significant and large effect on evaluations of Trump, *F* (1, 173) = 118.910, *p* < 0.001, ηρ^2^ = 0.417, with Trump supporters evaluating his competence (*M* = 22.47; SE = 0.70) as significantly higher than Clinton supporters (*M* = 11.62; SE = 0.71). When two-way interactions were considered, we found no significant effects for order × treatment, *F* (1, 173) = 0.245, *p* = 0.621, ηρ^2^ = 0.001, order × vote, *F* (1, 173) = 0.002, *p* = 0.962, ηρ^2^ < 0.001, or for treatment × vote, *F* (1, 173) = 0.018, *p* = 0.893, ηρ^2^ < 0.001. Likewise, the three-way interaction of order × treatment × vote, *F* (1, 173) = 0.288, *p* = 0.592, ηρ^2^ = 0.002, was not significant.

Consideration of participant perceptions of Trump’s trustworthiness suggest that the microexpression treatment did not have a significant effect, *F* (1, 173) = 1.144, *p* = 0.286, ηρ^2^ = 0.007. However, the order of presentation did have a significant and small-to-moderate effect on participant perceptions, *F* (1, 173) = 4.047, *p* = 0.046, ηρ^2^ = 0.024, with viewers seeing the Clinton video first perceiving Trump as having lower levels of trustworthiness (*M* = 12.63; SE = 0.83) than those viewing him first (*M* = 14.97; SE = 0.81). The interaction of these two variables, as was the case with perceptions of Trump’s competence, was not significant, *F* (1, 173) = 0.249, *p* = 0.619, ηρ^2^ = 0.001, nor were the interactions between order × vote, *F* (1, 173) = 0.951, *p* = 0.331, ηρ^2^ < 0.001, treatment × vote, *F* (1, 173) = 0.044, *p* = 0.834, ηρ^2^ < 0.001, or the three-way interaction of order × treatment × vote, *F* (1, 173) = 0.676, *p* = 0.412, ηρ^2^ = 0.004.

On the other hand, vote intention was highly significant and had a large effect, *F* (1, 173) = 151.624, *p* < 0.001, ηρ^2^ = 0.477, with Trump supporters evaluating his trustworthiness (*M* = 20.95; SE = 0.82) as much higher than Clinton supporters (*M* = 6.66; SE = 0.83).

## Study 2 Discussion

Findings from the replication and extension of Study 1 with a national sample of Trump and Clinton voters just prior to the election shows that the micro-expressions of fear did not in this instance affect respondents’ trait evaluations of Donald Trump. While the robust findings of Study 1 would lead one to consider the findings of Study 2 slightly unexpected, these results may be seen as a result of the highly politicized, contested, and even sensational presidential race. Specifically, while doubts and misgivings about both candidates were accentuated throughout the campaign, immediately prior to Study 1 being carried out, Trump had to address the infamous leaked “locker room talk” between him and Billy Bush that detailed his sexual assaults. However, the pool of respondents for Study 2, chosen based upon their being registered to vote and prospectively voting for either Trump or Clinton, most likely had made up their minds concerning both candidates and were unlikely to change their positions or opinions on candidate traits based upon contemporaneous information (Lodge and Taber, 2013). Thus, while both studies evidenced rather similar evaluations on both traits of competence and trustworthiness, and likewise vote intent for both studies had particularly large effect sizes on these measures, especially in comparison with that of the micro-expression treatment, the changing contextual information most probably affected participant interpretation of non-verbal communication by the candidates.

Thus, while trait attributions may be seen as relatively stable, research suggests perceptions of these traits can and do change over the course of a political campaign. Attitudes and feelings toward presidential candidates shift from the primary to general election season (Sullivan and Masters, 1988), with recent research suggesting a more direct role played by media coverage (Eberl et al., 2016). Specifically, given the cascading amount of media coverage during the campaign, including the “locker room talk” video, the debates, and the extensive use of social media by Trump that was then elaborated upon by the mass media, it was difficult not to develop a firmly held opinion on the candidates in the immediate lead up to the election.

## General Discussion

This study extends the literature on micro-expressions by considering how they affect trait perceptions (Svetieva and Frank, 2016), and specifically as to how they apply to the realm of politics (Stewart et al., 2009b). Previous research considering political figures considered historically relevant micro-expression stimuli, in this case President George H.W. Bush’s rally speech at the start of the first Iraq War (Stewart et al., 2009b), the lack of much prior research was due in great part to the rarity of these displays occurring. The direct and independent replication of Stewart et al. (2009b) likely was due to the decades that had elapsed between the original event and the experimental study (Brand, 2012). Furthermore, participant pool likely affects how non-verbal behavior is processed and appraised, with the potential for international onlookers to be differentially influenced by micro-expressions and other non-verbal behavior (D’Errico, 2019). Because the studies carried out here are contemporaneous with the electoral cycle in which the micro-expression stimuli used occurred, and involve participants that are personally invested in the outcome of the election, the studies are arguably conceptual replications due to changing political circumstances.

Perhaps most important for future research, the research carried out here emphasizes the importance of context on the impact of candidate micro-expressions, such as their opposing candidate, and the stage of the election cycle. As noted by the Dartmouth Group over a quarter of a century ago (McHugo et al., 1985; Masters et al., 1986), not only is the competition for leadership a very different situation from the act of engaging in leadership, with attitudes toward candidates evolving and consolidating over the course of a campaign (Sullivan and Masters, 1988), so too will the perceptions of candidates change and strengthen. For instance, recent research suggests that self-reports of participant responses to presidential candidates change at the conclusion of a campaign based upon the establishment of the winner and loser in the race (Stewart et al., 2020).

In the two studies carried out here, we find that not only does contemporaneous information, in the form of video presentation order, influence evaluative response of candidate traits, evidently so too does the point in the electoral cycle when the evaluations are made. While the different nature of the populations evaluated in Study 1 and Study 2 diminish the strength of inferences we can make, our findings suggest that micro-expressions and order of presentation influence trait perceptions of Donald Trump 3 weeks prior to the election, whereas only order of presentation influences these perceptions in the days immediately prior to Election Day. This in turn suggests that voter minds were largely made up regarding Trump’s traits (and Clinton’s) in the days immediately prior to votes were cast; at least enough so that the subtle micro-expressions did not have the hypothesized effect.

That does not mean that Trump’s micro-expressions did not have an impact upon the emotional state or perceptions of participants, just that it matters when evaluating candidate traits in a “high information” election that received unprecedented amounts of media coverage. Indeed, the influence of micro-expressions during a leader’s term of service is likely enhanced due to greater focus on the individual filling that role (Chance, 1967; Mazur, 2005). Furthermore, while previous research concerning micro-expressions has focused on their connection with deception and/or inappropriate emotional leakage, Study 1 finds they can positively influence trait attributions. This is especially the case when they are congruent with the verbal message, as was the case with Trump’s fear displays emphasizing the threat posed by terrorists. Indeed, such non-verbal emotional “leakage” or perhaps more appropriately “punctuation” as seen here apparently redounds to Trump’s benefit by underscoring his perceived authenticity. What may be occurring in the videos studied here is that the micro-expressions underscore the importance of the threat posed, providing Trump a more powerful “puzzle” for which he will provide the “solution” as president (Bull, 2003). In other words, Trump is often referred to by his supporters as “saying what he means and meaning what he says.” At the same time, it is important to note that Trump’s facial displays are only one piece of the non-verbal puzzle to understanding a man who is not easily studied via the verbal content of his utterances (e.g., D’Errico and Poggi, 2019; Kang et al., 2020).

The importance of the strength of emotional connection with the candidate has arguably been highlighted in an election where one candidate (Trump) won by strategically energizing his electorate, while the other (Clinton) lost by not applying the lessons long recognized by political psychologists–anxiety over the opposition does not get people to the polls; enthusiasm for a candidate does (Brader, 2006; Marcus, 2013). This was an election where authenticity and integrity was featured front and center in people’s support for a candidate, and each was evaluated more by the manner in which a candidate spoke rather than the factual accuracy of their verbal utterances. Indeed, if there is one defining lesson to be learned from this election, it is that the enthusiasm gap matters, and that non-verbal behavior plays a large role.

In trying to understand the ascendance of Donald Trump to the presidency, a potential issue facing political psychology specifically, and social sciences generally, is the tendency for academic research to focus on language-based analyses. Supporters perceive Donald Trump as meaning what he says, with his non-verbal behavior clearly connected with his verbal utterances; the question remains as to whether he says what he means. Namely, with his firmly stated yet ever-shifting policy positions, there is some doubt as to what is more reliable as an indicator of his positions–the cheap signals of the words he uses, or the costly signals of the non-verbal displays he shows and feels (Owren et al., 2010). While understanding verbal utterances of candidates such as Trump and the coverage of them via traditional media does provide insights into conventional politicians, it does not capture the emotional salience provided by non-verbal behavior, be it facial displays as studied here, or the body movements and vocalic utterances of the candidates. The emphasis on such efforts likely represent the relative ease of analyzing language through content analyses and self-report survey methodologies in comparison with visual and vocalic-focused studies, which rely on more resource intensive coding procedures and multi-method approaches to measuring voter perceptions and candidate impact.

Indeed, it should be noted that the studies carried out here used verbal scales to construct indices of trustworthiness and competence to evaluate non-verbal behavior; given the “halo effect” whereby all the favored candidate’s qualities are exaggerated and an often concurrent “devil shift” with the opposition candidate, these verbal scales likely are less useful the closer to election day they are taken with more heuristic processing based upon leadership prototype expectations taking hold (Trichas et al., 2017). Furthermore, the high level of media exposure during the 2016 presidential campaign, may have set in campaign fatigue. Regardless, the results reported here may be seen as an extension of the Dartmouth Group’s findings regarding the 1984 presidential election, with changing response to presidential candidate displays (Sullivan and Masters, 1988), and more generally a vindication for Fridlund’s insights concerning the role played by context on the evaluation of facial display behavior (Fridlund, 1994, 2017).

Furthermore, recent insights regarding emotion and its interpretation point to the difficulty in relying on its verbal definition as the semantics of emotion are introspective, subjective, and thus prone to a distribution across the population being studied (Barrett, 2017); as a result, it may be considered a separate behavior altogether (de Gelder, 2017; Patterson, 2017). Thus, future research should not only consider the larger socio-political context but also measure individual behavior in response to political leaders as directly as possible (e.g., ethologically and psycho-physiologically). Certainly cultural differences, especially for those living in non-WEIRD (Western, Educated, Individualistic, Rich, and Democratic) societies, will play a role in both the encoding and appraisal of facial display behaviors (Rychlowska et al., 2015) as will gender of those sending and receiving these displays (Brody and Hall, 2008). However, due to the nature of this research, which was bound by national context and affected by time and financial constraints, we were not able to recruit either a comparison group, nor attain the statistical power necessary to probe whether gender played a significant role in appraisal. We expect that, given the greater emphasis placed upon mediated communication in society, and worldwide, that such studies will be forthcoming.

Despite such shortcomings, the studies carried out here help us better appreciate the complexity of scientific research carried out “in the wild of politics.” Understanding our political leaders and shapers of the social lives of nations means appreciating the influence of the multiple characteristics of non-verbal communication pointed out by Patterson (2017)–components and patterns, determinants, and functions–and the role they play in the influencing followers. The importance of this is underscored by political leaders who now directly engage their followers in a virtual personal face-to-face relationship through the now near omnipresent social media that permeates all our lives.

## Data Availability Statement

The raw data supporting the conclusions of this article will be made available by the authors, without undue reservation.

## Ethics Statement

The studies involving human participants were reviewed and approved by University of Arkansas, Fayetteville. The patients/participants provided their written informed consent to participate in this study.

## Author Contributions

PS developed the study concept, drafted the manuscript, edited, programmed, and collected data in studies 1 and 2. ES contributed to critical editorial revisions. Both authors contributed to study design with both choosing and FACS coding stimuli materials, did data analysis, and approved the manuscript for submission.

## Conflict of Interest

The authors declare that the research was conducted in the absence of any commercial or financial relationships that could be construed as a potential conflict of interest.

## Footnotes

1 Although it should be noted that the ME-control video, with its more disjointed presentation, trended toward significant differences from the ME-intact (*p* = 0.112) and ME-removed (*p* = 0.163) conditions.

## Supplementary Material

Supplementary Figure 1 Donald Trump’s first micro-expression with lip corners stretching and lips parting (*20C [frames 1029-1046]* + *25B [frames 1025-1046]*).
