# Supplementary figures and images for: Micro-Expressions of Fear During the 2016 Presidential Campaign Trail: Their Influence on Trait Perceptions of Donald Trump
Source: Front Psychol. 2021 Jun 2;12:608483. doi: 10.3389/fpsyg.2021.608483 (PMC8206780; doi:10.3389/fpsyg.2021.608483)

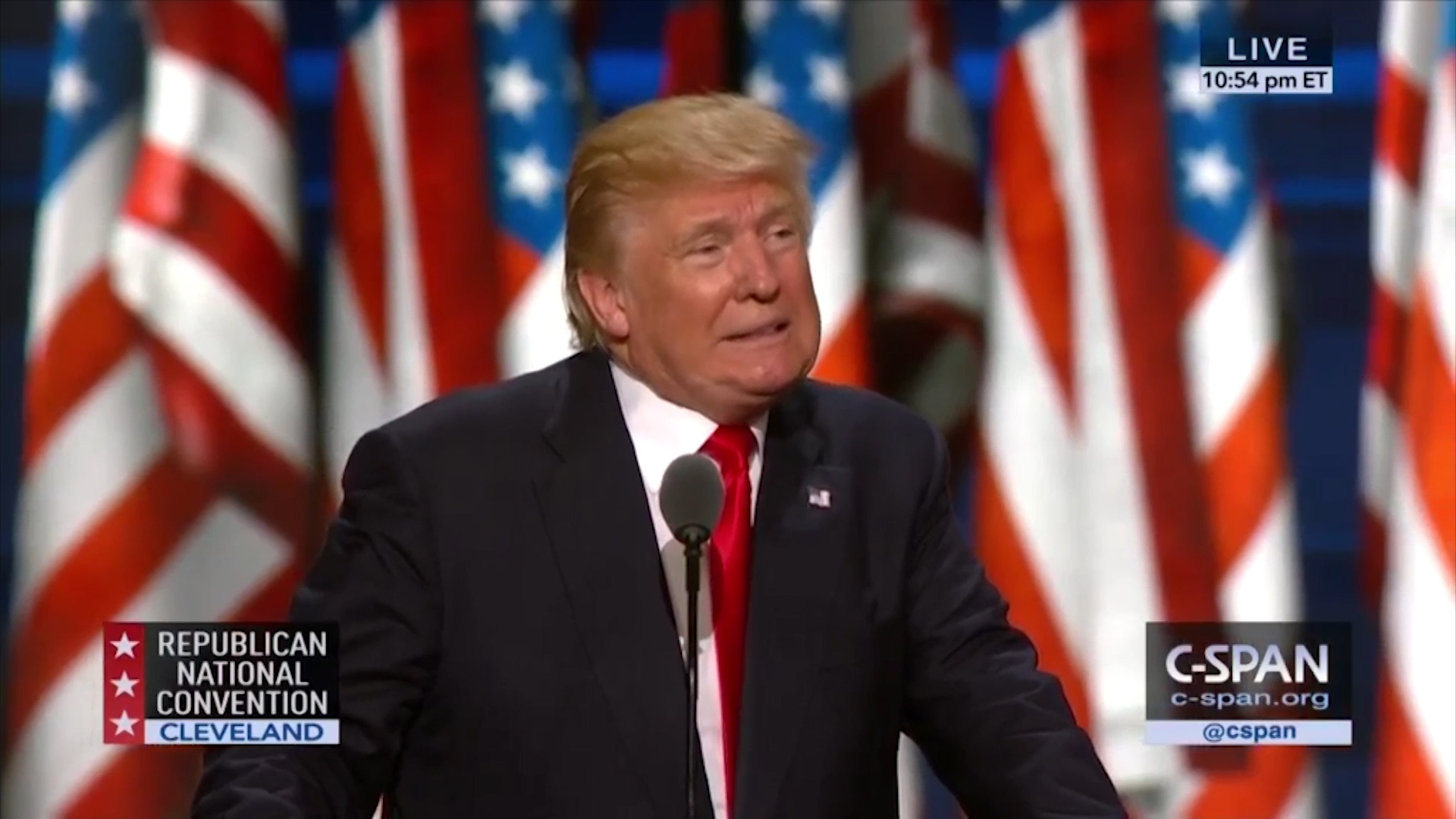

Supplement: Supplementary Figure 1 — Donald Trump’s first micro-expression with lip corners stretching and lips parting (20C [frames 1029-1046] + 25B [frames 1025-1046]). [file Image_1.JPEG]
